# The H_2_S Donor GYY4137 Stimulates Reactive Oxygen Species Generation in BV2 Cells While Suppressing the Secretion of TNF and Nitric Oxide

**DOI:** 10.3390/molecules23112966

**Published:** 2018-11-14

**Authors:** Milica Lazarević, Emanuela Mazzon, Miljana Momčilović, Maria Sofia Basile, Giuseppe Colletti, Maria Cristina Petralia, Placido Bramanti, Ferdinando Nicoletti, Đorđe Miljković

**Affiliations:** 1 Department of Immunology, Institute for Biological Research “Siniša Stanković”, University of Belgrade, Bulevar despota Stefana 142, 11060 Belgrade, Serbia; milica.laza93@gmail.com (M.L.); mommilja@yahoo.com (M.M.); sofiabasile@hotmail.it (M.S.B.); georgije_zw@yahoo.com (Đ.M.); 2 IRCCS Centro Neurolesi Bonino Pulejo, Strada Statale 113, C.da Casazza, 98124 Messina, Italy; emanuela.mazzon@irccsme.it (E.M.); m.cristinapetralia@gmail.com (M.C.P); placido.bramanti@irccsme.it (P.B.); 3 Department of Biomedical and Biotechnological Sciences, University of Catania, Via S. Sofia 89, 95123 Catania, Italy; Giuseppecolletti140@hotmail.com

**Keywords:** hydrogen sulfide, GYY4137, microglia, reactive oxygen species, inflammation

## Abstract

GYY4137 is a hydrogen sulfide (H_2_S) donor that has been shown to act in an anti-inflammatory manner in vitro and in vivo. Microglial cells are among the major players in immunoinflammatory, degenerative, and neoplastic disorders of the central nervous system, including multiple sclerosis, Parkinson’s disease, Alzheimer’s disease, and glioblastoma multiforme. So far, the effects of GYY4137 on microglial cells have not been thoroughly investigated. In this study, BV2 microglial cells were stimulated with interferon-gamma and lipopolysaccharide and treated with GYY4137. The agent did not influence the viability of BV2 cells in concentrations up to 200 μM. It inhibited tumor necrosis factor but not interleukin-6 production. Expression of CD40 and CD86 were reduced under the influence of the donor. The phagocytic ability of BV2 cells and nitric oxide production were also affected by the agent. Surprisingly, GYY4137 upregulated generation of reactive oxygen species (ROS) by BV2 cells. The effect was mimicked by another H_2_S donor, Na_2_S, and it was not reproduced in macrophages. Our results demonstrate that GYY4137 downregulates inflammatory properties of BV2 cells but increases their ability to generate ROS. Further investigation of this unexpected phenomenon is warranted.

## 1. Introduction

Hydrogen sulfide (H_2_S) is a gaseous signaling molecule that has anti-inflammatory and cytoprotective properties [1]. H_2_S is endogenously synthesized from l-cysteine in reactions catalyzed by cystathionine beta-synthase (CBS, EC 4.2.1.22) and gamma-cystathionase (CSE, EC 4.4.1.1) [2].

It has been suggested that H_2_S exerts neuroprotective effects in neuroinflammatory diseases, including neurodegenerative diseases, such as Parkinson’s disease, Alzheimer’s disease, and stroke, and autoimmune diseases of the central nervous system (CNS), such as multiple sclerosis [3,4,5].

More recently, a role for H_2_S has also been hypothesized in glioblastoma multiforme (GBM) and neuroblastoma in light of the finding that the expression and activity of CBS, gamma-cystathionase (CTH, EC 4.4.1.1), and 3-mercaptopyruvate sulfurtransferase (MPST, EC 2.8.1.2) were found in the human glioblastoma-astrocytoma (U-87 MG) and neuroblastoma (SHSY5Y) cell lines [6]. In both cell lines, the expression and activity of MPST was the highest among the investigated enzymes, suggesting its possible role in the generation of H_2_S. The higher expression and activity of CBS, CTH, and MPST in the neuroblastoma cells were associated with more intensive generation of H_2_S in the presence of 2 mM of cysteine. A threefold higher level of sulfane sulfur, a potential source of H_2_S, was detected in the astrocytoma cells in comparison to the neuroblastoma cells [6].

Accordingly, it has been proposed that H_2_S donors might be used as therapeutics for brain diseases [7]. Various H_2_S donors have been used in biomedical studies investigating the role of H_2_S in homeostasis and various pathologies [8]. While NaHS has been predominantly used in the studies, GYY4137 is a slow-releasing H_2_S donor that has been increasingly employed over the past 10 years. GYY4137 is a water-soluble derivative of Lawesson’s reagent that releases H_2_S via hydrolysis [8].

Microglia play a central role in the pathogenesis of various brain diseases, including neurodegenerative and autoimmune disorders and brain cancers [9,10]. In the steady state microglial cells largely contribute to the homeostasis of the CNS [11]. However, under excessive pro-inflammatory activation, they become highly pro-inflammatory and can contribute to autoimmune, degenerative, and neoplastic disorders of the CNS [9]. Activated microglia release various inflammatory and neurotoxic molecules that destroy the CNS tissue and contribute to various neurological disorders [9,10]. Microglial cells are able to perform phagocytosis and to present antigens to T cells through expression of major histocompatibility complex (MHC) molecules and costimulatory molecules, such as cluster of differentiation (CD)80 and CD40 [10]. Through release of inflammatory cytokines, such as tumor necrosis factor (TNF), interleukin (IL)-6 and IL-1β, and of nitric oxide (NO) and reactive oxygen species (ROS), they contribute to the pathology of various brain diseases, such as Parkinson’s disease, Alzheimer’s disease, or multiple sclerosis [9,10]. Regarding the role of glial cells in CNS oncogenesis, we and others have shown that dysregulated production of pro-inflammatory cytokines such as macrophage migration inhibitory factor (MIF) from glial cells and in the tumor microenvironment may contribute to the development, maintenance, and aggressiveness of GBM [12,13,14]. Therefore, regulation of microglia function may represent an important therapeutic target for the treatment of these disorders [9,11].

Along this line of research, we studied here the effects of GYY4137 on BV2 microglial cells. Reduction of TNF and NO release and downregulation of expression of antigen-presenting molecules, but upregulation of ROS generation, was observed under the influence of GYY4137. The obtained results are discussed in relation to the potential use of H_2_S donors as therapeutics for CNS diseases.

## 2. Results

### 2.1. GYY4137 Inhibits Production of NO and TNF in BV2 Cells

**Figure 1:**
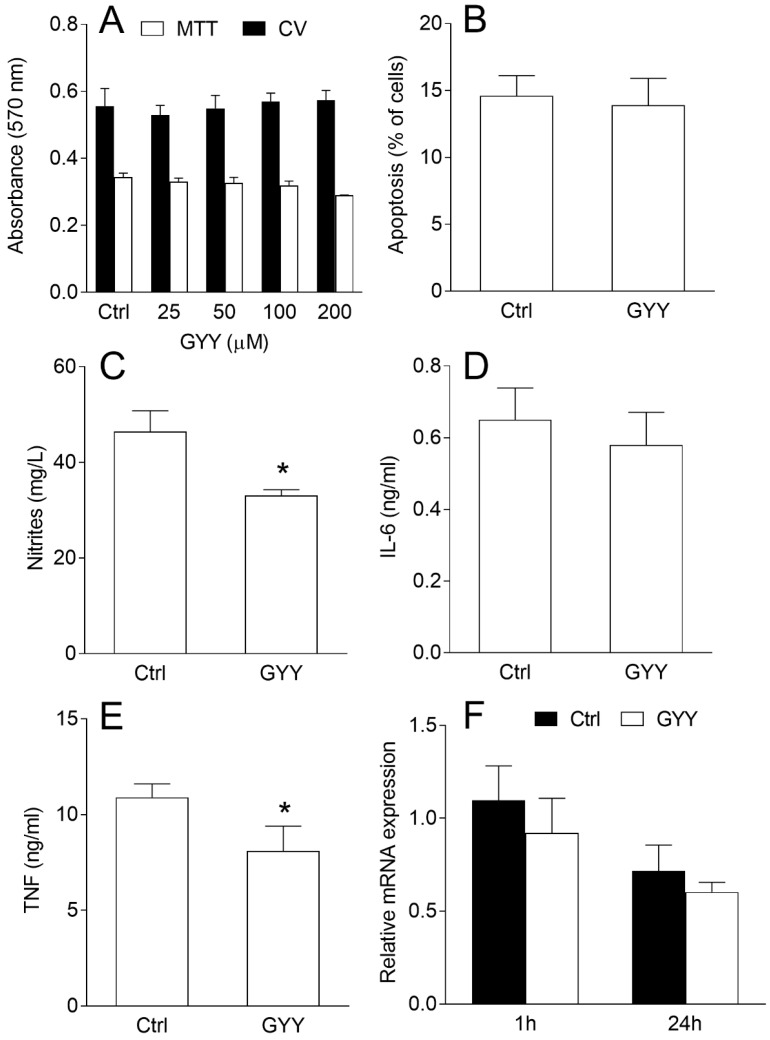
Effects of GYY4137 (GYY) on viability and nitric oxide (NO) and cytokine production in BV2 cells. BV2 cells were stimulated with interferon-γ (IFN-γ) and lipopolysaccharide (LPS) and cultivated in the presence of dimethyl sulfoxide (DMSO) (control—Ctrl) as the vehicle or GYY (200 μM in **B**–**F**) for 24 h. Subsequently, the cell viability was determined by crystal violet (CV) and 3-(4,5-dimethylthiazol-2-yl)-2,5-diphenyltetrazolium bromide (MTT) tests (**A**), apoptosis was detected by annexin V staining and cytofluorimetry (**B**), nitrites were measured by Griess reaction (**C**), cytokine concentrations were determined by ELISA (**D**,**E**), and mRNA expression relative to β-actin by real-time RT-PCR (**F**). Data are presented as mean + standard deviation (SD) from at least three independent experiments. * *p* < 0.05 refers to Ctrl.

BV2 cells were stimulated with interferon-γ (IFN-γ) and lipopolysaccharide (LPS) and exposed to various concentrations of GYY4137. There was no effect on BV2 cell viability after 24 h of cultivation even with the highest concentration applied (200 μM), as deduced by crystal violet (CV) and 3-(4,5-dimethylthiazol-2-yl)-2,5-diphenyltetrazolium bromide (MTT) tests (Figure 1A). The result was corroborated with annexin V staining, as there was no change in the percentage of apoptotic BV2 cells after the treatment with 200 μM of GYY4137 (Figure 1B). At the same time, levels of nitrites, as the measure of NO production, were reduced in the supernatants of GYY4137-treated cultures (Figure 1C). Similarly, TNF but not IL-6 levels in the supernatants were also reduced under the influence of GYY4137 (Figure 1D,E). Interestingly, the effects on TNF production were not paralleled with the inhibition of its RNA expression (Figure 1F).

### 2.2. GYY4137 Influences BV2 Cell Phenotype and Phagocytic Ability

**Figure 2:**
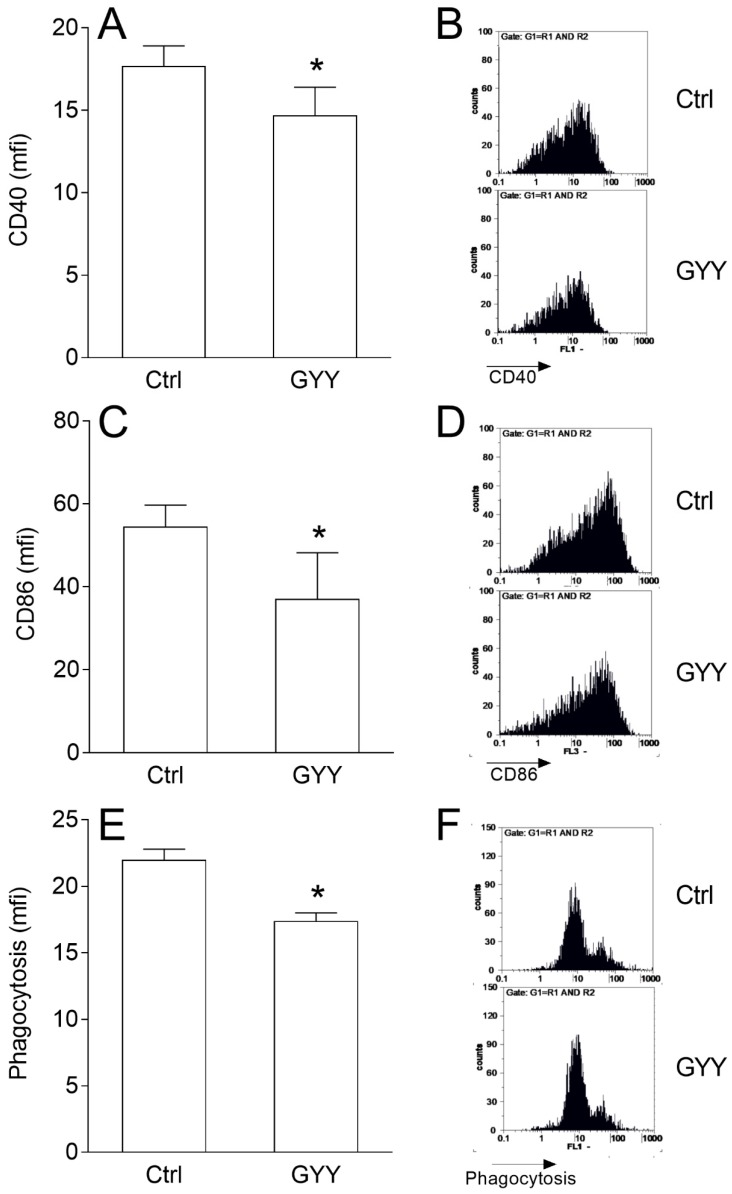
Effects of GYY4137 (GYY) on phenotype and phagocytosis in BV2 cells. BV2 cells were stimulated with IFN-γ and LPS and cultivated in the presence of DMSO (Ctrl) as the vehicle or GYY (200 μM) for 24 h. Subsequently, expression of CD40 (**A**,**B**), CD86 (**C**,**D**), and phagocytosis (**E**,**F**) were determined by cytofluorimetry. Data are presented as mean + SD from at least three independent experiments (**A**,**C**,**E**). Representative plots are also provided (**B**,**D**,**F**). * *p* < 0.05 refers to Ctrl.

In order to determine the effects of GYY4137 on the antigen-presenting function of BV2 cells, expression of costimulatory molecules CD86 and CD40 and phagocytosis of latex beads were determined. GYY4137 decreased expression of both molecules on BV2 cells (Figure 2A–D). It also inhibited phagocytosis of the latex beads by BV2 cells (Figure 2E,F).

### 2.3. GYY4137 Upregulates ROS Generation in BV2 Cells

**Figure 3:**
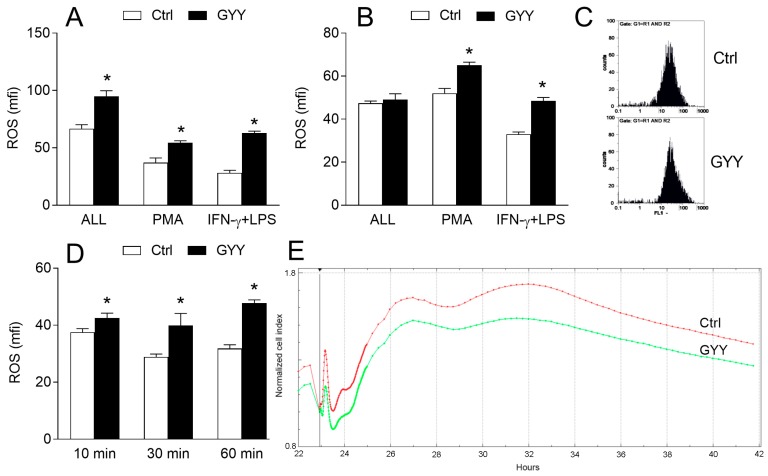

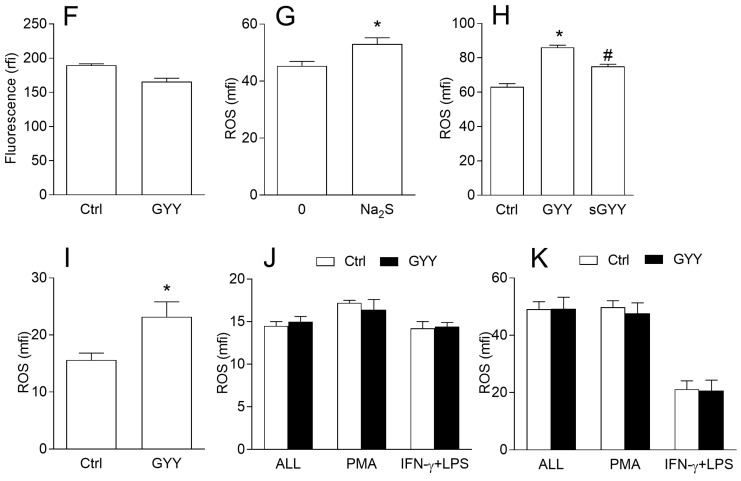
Effects of GYY4137 (GYY) on reactive oxygen species (ROS) production in BV2 cells. BV2 cells were cultivated in the presence of DMSO (Ctrl) as the vehicle or GYY (200 μM). (**A**) BV2 cells were treated with IFN-γ and LPS and DMSO or GYY for 24 h, stained with DHR, and stimulated with phorbol 12-myristate 13-acetate (PMA) for 90 min (ALL), or they were treated with DMSO or GYY for 24 h, stained with DHR, and stimulated with PMA or IFN-γ and LPS (IFN-γ + LPS) for 90 min. Subsequently, cytofluorimetry was performed. (**B**) Same as A, except that incubation lasted for 1 h instead of 24 h. (**C**) Representative DHR plots from (**B)**. (**D**) BV2 cells were treated with DMSO or GYY for the indicated time periods, stained with DHR, stimulated with PMA, and cytofluorimetry was performed. (**E**) BV2 cells were treated with IFN-γ and LPS and DMSO or GYY and analyzed by the real-time cell analyzer. Representative plot is provided. (**F**) DMSO or GYY were mixed with DHR in a cell-free system and fluorescence was detected on a fluorimeter. (**G**) BV2 cells were treated with Na2S (200 μM) or were untreated (0) for 60 min, stained with DHR, and stimulated with PMA for 90 min. Subsequently, cytofluorimetry was performed. (**H**) BV2 cells were treated with DMSO or GYY for 60 min, stained with DCFDA, and stimulated with PMA for 90 min. Subsequently, cytofluorimetry was performed. (**H**) Same as B (PMA), with additional treatment with “spent” GYY4137 (sGYY). (**I**) same as B (PMA), except that BV2 cells were stained with DCFDA (**J**). Same as B, except that mouse macrophages were used. (**K**) Same as B, except that rat macrophages were used. Data are presented as mean + SD from at least three independent experiments. * *p* < 0.05 refers to Ctrl or 0; ^#^ *p* < 0.05 refers to difference between GYY and sGYY.

BV2 cells were incubated with GYY4137 for different time periods, ranging from 10 min to 24 h, in the presence or absence of IFN-γ and LPS, and then they were stained with dihydrorhodamine 123 (DHR) and treated with phorbol 12-myristate 13-acetate (PMA) or IFN-γ and LPS to stimulate ROS generation. GYY4137 increased ROS production, as indicated by DHR fluorescence intensity, in BV2 cells in all cases (Figure 3A–D), even after only 10 min of incubation. The ability of GYY4137 to act quickly upon BV2 cells was confirmed by real-time cell analysis, where it was shown that GYY4137 acted in the very first minutes of its application, and that it also had a prolonged effect on BV2 cells (Figure 3E). To exclude the possibility that GYY4137 interacted with DHR regardless of BV2 cells, DHR and GYY4137 were co-incubated in a cell free system and fluorescence was detected by a fluorometer. No GYY4137-imposed increase in fluorescence was observed in the cell-free system (Figure 3F). The effects of GYY4137 were mimicked by another H_2_S donor, Na2S (Figure 3G), thus supporting the idea that increase of ROS in BV2 cells under the influence of GYY4137 was H_2_S dependent. This was further corroborated with the fact that GYY4137 that was incubated at 37 °C for 4 days before being used for the treatment of BV2 cells (“spent GYY”) partially lost its ROS-inducing activity (Figure 3H). In order to further test the results obtained with DHR, BV2 cells were stained with the other ROS detector, 2′,7′-Dichlorofluorescin diacetate (DCFDA), and an increase of ROS generation under the influence of GYY4137 was detected once again (Figure 3I). Finally, the effects of GYY4137 on ROS generation in macrophages were explored. No increase in ROS production in macrophages under the influence of GYY4137 was observed (Figure 3J,K), hence suggesting microglia-specific ROS-increasing effects of GYY4137.

## 3. Discussion

We have shown here that the H_2_S donor GYY4137 exerted pleiotropic effects on BV2 cells in vitro that included inhibitory effects on NO and TNF generation and expression of CD40 and CD86 along with clear-cut stimulation of ROS production.

The eventual significance of these effects of GYY4137 on BV2 cells in neurological pathologies, if any, must be proved in adequate in vivo studies. The inhibitory effects of GYY4137 on NO and TNF production may be beneficial in neuroimmunological diseases such as multiple sclerosis and neurodegenerative disorders, as these molecules are implicated as important mediators in their pathogenesis [15,16]. Also, downregulation of costimulatory molecules CD86 and CD40, which are important for efficient antigen presentation by microglial cells and are elevated in these cells during autoimmune events in the CNS [17], could be beneficial in CNS-directed autoimmune responses. The effects of H_2_S donors on BV2 cells have been previously studied [18]. In agreement with our data, GYY4137 inhibited LPS-induced NO generation both in BV2 cells and in the macrophage cell line RAW264.7 [18,19]. Differences in the effects of H_2_S on cytokine production in different monocytic cell lines have also been reported. In contrast to the report on RAW264.7 cells [19], it was shown that NaHS stimulated TNF, IL-6, and IL-1β in U937 monocytic cell lines [20]. The different experimental conditions used may also account for the divergent effects of GYY4137 on the TNF secretory capacity of RAW264.7 cells and U937 cells [19,20]. However, these data indicate that the effects of H_2_S donors on immune cells are divergent, and that in various settings, H_2_S activates or inhibits immune cells [2].

The observation of ROS potentiation in BV2 cells under the influence of GYY4137 was unexpected, as it was shown that H_2_S inhibits ROS generation in neuronal cells and protected cardiac cells against ROS effects [21,22,23,24]. Therefore, the ROS-potentiating effect of GYY4137 in BV2 cells was explored in numerous additional control experiments described in Section 2.3 that convergently support the finding that GYY4137 stimulates BV2 cells to generate ROS through H_2_S release. Although this phenomenon is unusual, it has been described previously in independent studies. It was shown that H_2_S-imposed hepatotoxicity is mediated through ROS induction [25]. H_2_S genotoxic effects on Chinese hamster ovary cells were also mediated through ROS [26]. Furthermore, H_2_S induced oxidative DNA and RNA damage in coelomocytes of marine invertebrates [27]. Interestingly, positive effects of H_2_S on ROS generation were also reported in plants, where H_2_S-stimulated H_2_O_2_ production in *Arabidopsis* guard cells was implicated in stomatal closure [28]. Finally, it was reported that H_2_S inhibited growth of *Escherichia coli* through induction of ROS [29]. Thus, ROS-mediated effects of H_2_S have been observed in evolutionarily distant organisms. The significance of upregulated production of ROS induced by H_2_S may warrant further investigations.

There are numerous reports on the beneficial effects of H_2_S in various brain pathologies. H_2_S protects neurons from cell death provoked by oxidative glutamate toxicity or anoxia/reoxygenation [30,31]. The neuroprotective effects of H_2_S donors were also observed in vivo [32,33,34,35]. Furthermore, NaHS or GYY4137 counteracted inflammation as well as oxidative and nitrosative stress in animal models of Alzheimer’s disease [36], Parkinson’s disease [37], high-salt-induced hypertension [38], and cerebral malaria [39]. Importantly, the protective effects of H_2_S in hypoxia-induced neurotoxicity in vivo were shown to be mediated through inhibition of microglial activation, including NO and cytokine generation [40]. Similarly, anti-inflammatory effects of NaHS in a repetitive febrile seizure model were paralleled with inhibition of microglial activity [41]. Moreover, endogenous H_2_S was shown to be of great importance for microglial shift from pro- to anti-inflammatory in cerebral ischemia [42].

Our results are in concordance with these data as far as NO and TNF are concerned. As discussed, the major discordant result is the observed potentiation of ROS by GYY4137 in BV2 cells. However, negative effects of H_2_S on brain pathology, such as cerebral ischemic damage, were also previously reported [43]. As it was postulated that cerebral ischemic damage is mediated through ROS [44,45], it would be important to examine if the damage-potentiating effects of H_2_S are ROS dependent. Also, caution should be exercised when considering ROS strictly as pro-inflammatory and damaging mediators. It is well known that both ROS and H_2_S are not only effector but also signaling molecules working through thiol-based and other redox switches [46]. H_2_S-mediated ROS generation might have regulatory rather than effector properties and it is, therefore, essential to test the observed effects of GYY4137 on microglial cells in various settings in which microglial cells interact with other cells.

On the other hand, the presently observed combined downregulation of NO and TNF along with the upregulation of ROS allows us to hypothesize that H_2_S donors may be useful for the treatment of + certain brain tumors such as GBM. In fact, it is known that the local production of pro-inflammatory cytokines such as MIF and TNF [47,48,49] in the tumor microenvironment of solid tumors such as GBM and melanoma induce the production of NO and other immunosuppressive mediators that may favor growth and invasiveness of GBM [12,13]. Vice versa, experimental chemotherapeutic drugs exert their action on the viability of GBM cell lines through local production of ROS and subsequent JNK activation [50,51].

Like the other endogenous gas NO, which has been indicated both as an oncogenic and oncosuppressor molecule, with its ultimate fate probably depending on concentration and time of exposure [52], conflicting data have been generated on the chemotherapeutic potential of H_2_S donors or H_2_S inducers. This observed pharmacological profile of GYY4137 in BV2 cells warrants additional in vivo studies of H_2_S and its donors and inducers also in the field of neuro-oncology [53,54].

Defining the eventual suitable area of application of H_2_S in CNS disorders would be of immediate utility for the clinical setting, as several H_2_S-releasing drugs, such as SG1002 for cardiovascular disorders and ATB-346 for arthritis, have progressed into clinical trials and could be tested in CNS pathologies of neuroinflammatory, neurodegenerative, or neoplastic origin once clear-cut proof of concepts have been generated in relevant animal models [55].

## 4. Materials and Methods

### 4.1. Cell Cultures and Reagents

Unless specifically stated, chemicals used in the experiments were from Sigma-Aldrich (St. Louis, MO, USA). BV2 cells were obtained as the kind gift of Dr. Alba Minelli, Università degli Studi di Perugia, Perugia, Italy. BV2 cells were grown and cultured in RPMI-1640 culture medium (Biological Industries, Kibbutz Beit-Haemek, Israel) that was supplemented with 5% heat-inactivated fetal calf serum (FCS, PAA Laboratories, Pasching, Austria) at 37 °C in a humidified atmosphere containing 5% CO_2_. For the treatments, BV2 cells were seeded at 5 × 10^5^/mL/well in 24-well plates (Sarstedt, Pasching, Austria). BV2 cells were stimulated with 10 ng/mL of recombinant mouse IFN-γ and 100 ng/mL of LPS and treated with various concentrations of GYY4137 (Cayman Chemical, Ann Arbor, MI, USA).

### 4.2. Viability Assays

Viability of BV2 was assessed by CV and MTT tests. At the end of the treatment, cells were washed with phosphate-buffered saline (PBS) to remove nonadherent dead cells. For the CV test, the remaining cells were fixed with methanol. After staining with 1% CV solution, the plates were thoroughly washed and then the dye was dissolved in 33% acetic acid. For the MTT assay, adherent BV2 cell were exposed to 0.5 μg/mL MTT solution and incubated for 30 min at 37 °C. Cell culture supernatants were removed and dimethyl sulfoxide (DMSO) was added to the plates to dissolve the formazan crystals. The absorbance of dissolved dyes, corresponding to the number of viable cells, was measured in triplicates at 540 nm with a correction at 670 nm using an automated microplate reader (LKB 5060-006, LKB, Vienna, Austria).

### 4.3. Detection of NO Release

Nitrite accumulation, a measure of NO release, was determined in cell culture supernatants using the Griess reaction. In brief, triplicate aliquots of cell-free supernatants were mixed with an equal volume of Griess reagent (1:1 mixture of 0.1% naphthylethylenediamine dihydrochloride and 1% sulphanilamide in 5% H_3_PO_4_). The absorbance at 540 nm was determined with a microplate reader and compared to a standard curve for NaNO_2_.

### 4.4. ELISA Test for Determination of Cytokines

Cytokine concentration in cell culture supernatants was determined by sandwich ELISA using MaxiSorp plates (Nunc, Roskilde, Denmark) and anticytokine paired antibodies according to the manufacturer’s instructions. Samples were analyzed in duplicates for murine TNF (eBioscience, San Diego, CA, USA) and murine IL-6 (Abcam, Cambridge, UK). The results were calculated using standard curves made on the basis of known concentrations of the appropriate recombinant cytokines.

### 4.5. Real-Time Reverse Transcription Polymerase Chain Reaction

Total RNA was isolated from cells using a mi-Total RNA Isolation Kit (Metabion, Martinsried, Germany) and reverse transcribed using random hexamer primers and MMLV (Moloney Murine Leukemia Virus) reverse transcriptase according to the manufacturer’s instructions (Fermentas, Vilnius, Lithuania). Prepared cDNAs were amplified by using Maxima SYBR Green/ROX qPCR Master Mix (Fermentas, Vilnius, Lithuania) according to the recommendations of the manufacturer in an ABI PRISM 7000 Sequence Detection System (Applied Biosystems, Foster City, CA, USA). Thermocycler conditions comprised an initial step at 50 °C for 5 min, followed by a step at 95 °C for 10 min, and a subsequent two-step PCR program at 95 °C for 15 s and 60 °C for 60 s for 40 cycles. The PCR primers (Metabion, Martinsried, Germany) were as follows: TNF: 5′-CCA CGT AGC AAA CCA C-3′; 5′-TGG GTG AGG AGC ACG TAG T-3′; (GenBank accession no. NM 013693.2) and β-actin: 5′-CCA GCG CAG CGA TAT CG-3′; 5′-GCT TCT TTG CAG CTC CTT CGT-3′; (GenBank acc. No. NM_007393.3). Accumulation of PCR products was detected in real time and the results were analyzed with 7500 System Software (Applied Biosystems, Foster City, CA, USA). Relative RNA expression is presented as 2−dCt, where dCt is the difference between Ct values of a gene of interest and the endogenous control (β-actin).

### 4.6. Cytofluorimetry

BV2 cells were stained with FITC-conjugated anti-CD40 (eBioscience, San Diego, CA, USA) and Pe-Cy5-conjugated anti-CD86 (eBioscience, San Diego, CA, USA). Appropriate isotype control antibodies were used where necessary to set gates for cell marker positivity. Typically, proportion of isotype control antibody-stained cells was <1%. Detection of apoptosis was performed via annexin V-FITC staining (Biotium, Hayward, CA, USA). Cells positive for annexin V-FITC were considered apoptotic. For detection of phagocytosis, BV2 cells were plated in 24-well plates at 1 × 10^5^/well and pretreated with GYY4137 for 24 h. Latex beads (1 μm, yellow-green) were preopsonized in PBS supplemented with 50% FCS. Medium was discarded and the preopsonized beads were added (10 beads per cell), and cells were incubated at 37 °C for an additional hour. BV2 cells were analyzed with a CyFlow Space flow cytometer (Partec, Munster, Germany). Results of cytofluorimetry are presented as the proportion of cells bound by an appropriate antibody or as mean fluorescent intensity (mfi) of cell population. Cells were gated on live cells (R1) according to FSC vs. SSC and on cell singlets (R2) according to FSC vs. FSC-width, and mfi (arithmetic mean) was determined for all the cells within G1 (R1 and R2) by FloMax software for cytometry (Partec, Munster, Germany).

### 4.7. Detection of Reactive Oxygen Species (ROS) Generation

For detection of ROS generation, DHR or DCFDA staining was performed. The cells were incubated in the presence of 5 μM of DHR or 5 μM of DCFDA for 20 min. BV2 cells were pre-incubated with GYY4137 in the presence of IFN-γ (10 ng/mL) and LPS (100 ng/mL) for the indicated time periods, stained with DHR, and then stimulated with PMA (100 ng/mL) for an additional 90 min. Alternatively, BV2 cells were pre-incubated with GYY4137 for the indicated time periods, stained with DHR or DCFDA, and then stimulated with PMA (100 ng/mL) or IFN-γ (10 ng/mL) and LPS (100 ng/mL). The fluorescence was acquired via flow cytometry.

### 4.8. Real-Time Cell Analysis

Cell activation was characterized in real time by using an xCELLigence RTCA DP analyzer (Roche Diagnostics, Pleasanton, CA, USA). BV2 cells were seeded at 2.5 × 10^4^ cells into each well of an E-Plate 16 (Roche Diagnostics, Pleasanton, CA, USA). After 24 h of initial adherence, BV2 cells were treated with IFN-γ (10 ng/mL) and LPS (100 ng/mL) and GYY4137 (200 μM) or DMSO (solvent control). Impedance measurements were recorded every 15 s during the first 2 h of cultivation and then every 15 min overnight. Impedance measurements were recorded as a combined cellular index of proliferation, viability, activation, and morphology changes.

### 4.9. Statistical Analysis

Data were analyzed using two-way ANOVA followed by Tukey’s test or by Student’s *t*-test, as appropriate. A *p*-value less than 0.05 was considered statistically significant.
